# Local Application of Gentamicin in the Prophylaxis of Perineal Wound Infection After Abdominoperineal Resection: A Systematic Review

**DOI:** 10.1007/s00268-015-3159-5

**Published:** 2015-07-14

**Authors:** G. D. Musters, J. W. A. Burger, C. J. Buskens, W. A. Bemelman, P. J. Tanis

**Affiliations:** Department of Surgery, Academic Medical Center, University of Amsterdam, Post box 22660, 1105 AZ Amsterdam, The Netherlands; Department of Surgery, Erasmus Medical Center/Daniel den Hoed, Post box 5201, 3008 AE Rotterdam, The Netherlands

## Abstract

**Background:**

Use of topical antibiotics to improve perineal wound healing after abdominoperineal resection (APR) is controversial. The aim of this systematic review was to determine the impact of local application of gentamicin on perineal wound healing after APR.

**Methods:**

The electronic databases Pubmed, EMBASE, and Cochrane library were searched in January 2015. Perineal wound outcome was categorized as infectious complications, non-infectious complications, and primary perineal wound healing.

**Results:**

From a total of 582 articles, eight studies published between 1988 and 2012 were included: four randomized controlled trials (RCTs), three comparative cohort studies, and one cohort study without control group. Gentamicin was administered using sponges (*n* = 3), beads (*n* = 4), and by local injection (*n* = 1). There was substantial heterogeneity regarding underlying disease, definition of outcome parameters and timing of perineal wound evaluation among the included studies, which precluded meta-analysis with pooling. Regarding infectious complications, three of six evaluable studies demonstrated a positive effect of local application of gentamicin: one of four RCTs and both comparative cohort studies. Only two RCTs reported on non-infectious complications, showing no significant impact of gentamicin sponge. All three comparative cohort studies demonstrated a significantly higher percentage of primary perineal wound healing after local application of gentamicin beads, but only one out of three evaluable RCTs did show a positive effect of gentamicin sponges.

**Conclusion:**

Currently available evidence does not support perineal gentamicin application after APR.

## Introduction

Impaired perineal wound healing after abdominoperineal resection (APR) is a clinically significant problem. Perineal wound infection, wound dehiscence, and deep pelvic abscess are frequently encountered complications, which often require intensive and long-lasting wound care and interfere with quality of life. Furthermore, modified approaches for distal rectal cancer have increased the incidence of perineal wound problems after APR, especially the use of neo-adjuvant radiotherapy [1]. Surgical site infections, like perineal wound infection, may result in prolonged hospitalization, readmission, increase in health care costs, and even an increase in mortality [2]. The cornerstone in the prevention of wound infection is the use of systemic and/or oral prophylactic antibiotics. Despite prophylactic antibiotics, perineal wound infection has been reported to occur in more than half of the patients who undergo an APR after neo-adjuvant radiotherapy for rectal cancer [3]. For the antibiotics to be effective, a high antibiotic concentration at the designated site is warranted. However, systemic administration hardly effects perineal wound healing. This is most likely due to dead space with retention of fluids and surgically impaired blood supply. Perineal application leads to high local concentrations with low serum levels and hence low systemic adverse reactions [4]. In general, local gentamicin application is regarded as safe, easy to use, and inexpensive [5]. However, there is still no consensus on the clinical value of topical gentamicin to improve perineal wound healing in patients undergoing APR. Therefore, we conducted a systematic review of the literature on the effect of local gentamicin in preventing perineal wound infection and improving perineal wound healing after APR.

## Methods

### Search strategy

All studies evaluating the impact of local application of gentamicin on perineal wound healing after APR were considered eligible. Inclusion was not restricted to study design (i.e., randomized controlled trial (RCT), prospective or retrospective cohort study (with or without control group), and underlying disease (i.e., rectal cancer, anal cancer, and inflammatory bowel disease). Animal studies, systematic reviews, non-English articles, case series (<5 patients), and congress abstracts were excluded. Studies were identified by searching electronic databases and scanning of reference lists. This search was applied in PubMed (1971–2015), EMBASE (1982–2015), and Cochrane library (2005) databases in January 2015. The following medical subject heading (MESH) terms were used; gentamicin, aminoglycosides, anti-bacterial agents, perineum, abdomen, colorectal surgery, surgical procedures operative, and general surgery. Other search terms were abdominal perineal resection, abdominoperineal resection, abdominoperineal excision, abdominal perineal excision, perineum surgery, perineal surgery, gentamicin, gentamycin, aminoglycoside antibiotics, and local antibiotics. No language or publication date restrictions were applied. The candidate publication titles and abstracts were screened to exclude non-related publications. Secondly, the full text of the remaining manuscripts was read to determine whether they were eligible for inclusion.

### Validity assessment and assessment of eligibility

Methodological quality and risk of bias were assessed for the included studies. For cohort studies, the Newcastle Ottawa quality assessment scale for cohort studies was used to assess risk of bias [6]. The quality items scored were as follows: representativeness of the exposed cohort, selection of the non-exposed cohort, ascertainment of exposure, the absence of outcome of interest at the start of the study, validity of the design or analysis, assessment of outcome, duration of follow-up, and lost to follow-up. For RCTs, the Jadad scoring system was used to assess the risk of bias [7].

### Data extraction and data analysis

Data were extracted from the included studies by two independent investigators (GM, PT). Disagreements were resolved by discussion between the two reviewers. For each article, the following data were collected: year of publication, study design, number of patients, underlying disease, comorbidities, type of APR, neo-adjuvant radiotherapy, perineal closure technique, perineal drainage, systemic antibiotic use, administration method and dosage of locally applied gentamicin, definitions of outcome parameters, follow-up period, type and incidence of perineal wound complications, primary perineal wound healing rate, reoperation rate, and hospital stay. Meta-analysis was not intended because of the heterogeneity among studies regarding study population, variety in gentamicin application, and non-uniform definitions of outcome parameters.

## Results

The systematic search resulted in a total of 582 articles. After removal of duplicates among the different databases, 527 articles remained. After screening of publication titles and abstracts, 17 publications were retrieved for full text review. Of these 17 publications, 10 studies were excluded, because gentamicin was not placed in the perineal wound (*n* = 3) [8–10], no separate data for perineal and abdominal wound outcome were provided (*n* = 4) [11–14], surgery was not an APR procedure (*n* = 1) [15], use of bacitracin spray instead of gentamicin (*n* = 1) [16], and the type of local antibiotic was not described (*n* = 1) [17]. One additional study was included after crosschecking reference lists of the included studies. In total, eight studies were included of which four were RCTs, three were comparative cohort studies, and the remaining study was a non-comparative cohort study. After exclusion of patients who underwent non-APR surgery from two cohort studies [18, 19], a total number of 602 patients were included in this systematic review. The risk of bias of the included studies is displayed in “Appendix”.

### Patient characteristics

Patient characteristics of each included study are displayed in Table 1. APR was performed for cancer in 68 % (*n* = 381) of the patients, which was primary rectal cancer in 82 % (*n* = 311; five studies) [19–23], anal cancer in 4 % (*n* = 15; two studies) [20, 23], locally recurrent rectal or anal cancer in 1 % (*n* = 4; one study) [20], sarcoma in one patient [20], and unspecified type of cancer in 13 % (*n* = 50; two studies) of the patients [16, 24]. In three studies, patients with inflammatory bowel disease were also included with a specified number of patients in two studies (total *n* = 46) [18, 20, 24]. In the remaining patients, APR was performed for incontinence [20], villous adenoma [24], or unspecified diseases [18, 25]. Comorbidities related to risk of wound infection were described in two studies [21, 22]. Use of neo-adjuvant radiotherapy was described in three studies and was given to 21 % (*n* = 115) of the included patients [20–22]. Type of neo-adjuvant radiotherapy was only specified in one study (5 × 5 Gy) [21]. One study described prior pelvic radiotherapy in four patients [20].

**Table 1:** Study characteristics of comparative and non-comparative studies evaluating the impact of local gentamicin on perineal wound healing after APR

Included studies	Study design	Year	Local genta	*n*	Patients	Gender (male/female)	Underlying disease		Comorbidity		Radiotherapy	Intra-operative contamination	Type of surgery
First author					Age (years)		Cancer^a^ *n* (%)	IBD *n* (%)	Diabetes *n* (%)	Smoking *n* (%)	*n* (%)	*n* (%)	*n (%)*
Collin et al. [7]	RCT	2012	Yes	52	65 (29–87)^c^	32/20	39 (75)	13 (25)	–	–	37/39^e^ (94)	3 (6)	APR 38 (73) Intersphincteric APR 14 (27)
			No	50	67 (35–85)^c^	29/21	44 (88)	5 (10)	–	–	41/44^e^ (93)	8 (16)	APR 44 (88) Intersphincteric APR 6 (12)
De Bruin et al. [8]	Prospective comparative cohort study	2008	Yes	19	71 ± 10^b^	12/07	19 (100)	0	3 (16)	7 (37)	19 (100)	–	Standard APR 19 (100)
			No	21	69 ± 9^b^	13/08	21 (100)	0	5 (24)	8 (38)	21 (100)	–	Standard APR 21 (100)
Gruessner et al. [11]	RCT	2001	Yes	49	62 (44–83)^b^	–	49 (100)	0	4 (8)	14 (29)	1 (2)	–	Miles APR 49 (100)
			No	48	63(41–90)^b^	–	48 (100)	0	7 (15)	10 (21)	0	–	Miles APR 48 (100)
Rosen et al. [27]	RCT	1991	Yes	22	67 (44–84)^b^	11/11	22 (100)	0	–	–	–	–	Loyd-Davies APR 22 (100)
			No	22	64 (46–83)^b^	12/10	22 (100)	0	–	–	–	–	Loyd-Davies APR 22 (100)
Moesgaard et al. [17]	RCT	1988	Yes	41	54 (21–80)^c^	22/19	25 (61)	16 (39)	–	–	–	–	APR 34 (83) Proctocolectomy 7 (17)
			No	38	62 (17–83)^c^	19/19	25 (66)	12 (32)	–	–	–	–	APR 37 (97) Proctocolectomy 1 (3)
Sachweh et al. [30]	Retrospective comparative cohort study	1988	Yes	80	67^b, d^	–	–	–	–	–	–	–	Abdominal sacral rectum amputation 80 (100)
			No	26	64^b, d^	–	–	–	–	–	–	4 (15)	Abdominal sacral rectum amputation 26 (100)
Mühleder et al. [19]	Retrospective comparative cohort study	1988	Yes	67	–	–	67 (100)	0	–	–	–	–	APR 67 (100)
			No	42	–	–	42 (100)	0	–	–	–	–	APR 42 (100)
Lütje et al. [15]	Cohort study	1988	Yes	25	–	–	NS	NS	–	–	–	–	Abdominosacral proctectomy 24 (100)

*RCT* randomized controlled trial, *IBD* inflammatory bowel disease (Crohn’s disease and Ulcerative colitis), *APR* abdominoperineal resection, *NS* not specified

^a^Cancer: anal cancer, rectal cancer and recurrences

^b^Mean age ± standard deviation or (range)

^c^Median age (range)

^d^Distribution unknown

^e^This group was only calculated for cancer patients

### Surgery and prophylactic antibiotics

The different types of surgery being performed in the included studies are specified in Table 1. None of the studies described the use of an omental plasty. Primary perineal wound closure was performed in all studies, of which four studies mentioned closure in multiple layers [20–23]. Routine pelvic drainage was described in seven studies [18, 19, 21–25], and a perineal drain was selectively placed in one study [19]. Type of drainage differed among study groups in one study, with irrigation-suction drainage in the control group, and either continuous or intermittent drainage in the local gentamicin beads group [25].

Considering the type of local administration of gentamicin at the perineal wound, a collagen carrier was used in three studies [20–22], gentamicin beads were used in four studies [18, 19, 23, 25], and gentamicin was locally injected in one study (Table 2) [24]. Dosage of locally applied gentamicin was provided in four studies. A total dose of 210 mg [22], 200 mg [20], and 160 mg [24] was used in three studies. In the remaining study, the concentration of gentamicin was measured in the wound fluid with a mean value of 138.1 µg/l (*n* = 7, standard deviation 58.4) at day one post-operatively [23]. Four studies described placement of the local antibiotic in the sacral cavity [21–23, 25], while a more superficial application in the perineal wound was reported in the other four studies [18–20, 24]. Seven of eight studies described routine use of different regimens of prophylactic pre-operative antibiotics, and five studies also continued prophylactic antibiotics post-operatively, only in a subgroup of patients in one of these five studies (Table 2).

**Table 2:** Use of local and systemic antibiotics in patients undergoing APR with primary perineal closure

Included studies	*n*	Local antibiotics			Systemic antibiotics	
First author		Local (Yes/no)	Type	Location	Pre-operative	Post-operative
Collin et al. [7]	52	Yes	Genta sulfate sponge 2.0 mg/m^2^ (Collatamp)	Perineal wound	Prophylaxis not specified	Not specified *n* (%): 11 (21)
	50	No	–	–	Prophylaxis not specified	Not specified *n* (%): 11 (22)
De Bruin et al. [8]	19	Yes	Genta sponge (Garacol), 3 per patient	Sacral cavity	Augmentin 1 × 1000/200 mg	–
	21	No	–	–	Augmentin 1 × 1000/200 mg	–
Gruessner et al. [11]	49	Yes	Genta sponge (Septocoll), 3 per patient	Sacral cavity	Cefa 1 × 2 g, metro 1 × 500 mg	–
	48	No	–	–	Cefa 1 × 2 g, metro 1 × 500 mg	–
Rosen et al. [27]	22	Yes	Genta-PMMA, 30 beads, 1 chain per patient	Sacral cavity	Cefa 1 × 2 g, metro 1 × 500 mg	Cefa 2 × 2 g, metro 2 × 500 mg; day of operation
	22	No	–	–	Cefa 1 × 2 g, metro 1 × 500 mg	Cefa 2 × 2 g, metro 2 × 500 mg; day of operation
Moesgaard et al. [17]	41	Yes	Injection 160 mg genta/400 mg metro	Perineal wound	Genta 1 × 80 mg, metro 1 × 500 mg	Genta 3 × 80 mg, metro 3 × 500 mg; 2 days
	38	No	–	–	Genta 1 × 80 mg, metro 1 × 500 mg	Genta 3 × 80 mg, metro 3 × 500 mg; 2 days
Sachweh et al. [30]	80	Yes	Genta-PMMA, 30 beads, 2 chains per patient	Sacral cavity	–	–
	26	No	–	–	–	–
Mühleder et al. [19]	67	Yes	Genta-PMMA, 1 chain per patient	Perineal wound	Paro 4 × 500 mg, metro 3 × 500 mg orally	Metro 3 × 500 mg; 3 days
	42	No	–	–	–	–
Lütje et al. [15]	25	Yes	Genta PMMA 30-beads (Septopal), 1 chain per patient	Perineal wound	Latamoxef 1 × 2 g	Latamoxef 1 × 2 g 12 h after incision

*Cefa* cefazolin, *Metro* metronidazole, *Paro* paromomycin, *Genta* gentamicin

### Perineal wound outcome

Perineal wound outcome parameters were defined in six out of eight included studies, all being different among these six studies (Table 3). Time points for wound evaluation were provided in five studies, which were also not uniform.

**Table 3:** Perineal wound outcome after APR with primary perineal closure and local application of gentamicin

Included studies		Perineal wound evaluation	Time interval (s)	Study group		Perineal wound outcome			Perineal Reoperation	Hospital stay
First author	Study design	Definition outcome parameters		Type of genta	n	Infectious complications *n* (%)	Non-infectious complications *n* (%)	Primary healing *n* (%)	*n* (%)	Days
Collin et al. [7]	RCT	Infectious complications = redness, swelling and/or purulent discharge and/or open infected wound Non-infectious complication = open clean wound, persistent sinus/fistula Perineal wound healing = without infectious or non-infectious complication	7–10 days 1,3,12 months	Genta sponge	52	7 d: 6/52 (12) 1 m: 10/52 (19) 3 m: 3/51 (6) 12 m: 0/48 (0)	7 d: 2/52 (4) 1 m: 14/52 (27) 3 m: 12/51 (24) 12 m: 5/48 (10)	7 d: 43/52 (83) 1 m: 28/52 (54) 3 m: 36/51 (71) 12 m: 43/48 (90)	2 (4)	11 (6–52)^c^
				Control	50	7 d: 6/49 (12) 1 m: 14/49 (29) 3 m: 1/48 (2) 12 m: 0/43 (0)	7 d: 3/49 (6) 1 m: 12/49 (24) 3 m: 17/48 (35) 12 m: 5/43 (12)	7 d: 40/49 (82) 1 m: 23/49 (47) 3 m: 30/48 (63) 12 m: 38/43 (88)	3 (6)	10 (4–27)^c^
De Bruin et al. [8]	CCS	Superficial infection = cellulitis, no evidence of deep tissue infection Deep infection = skin and subcutaneous breakdown with infection + sacral/deep wound abscess	–	Genta sponge	19	Superficial: 2 (11)^*^ Deep: 1 (5)	–	16 (84)*	–	15 ± 8^*,d^
				Control	21	Superficial: 6 (29) Deep: 6 (29)	–	9 (43)	–	25 ± 18^d^
Gruessner et al. [11]	RCT	Primary wound healing = no signs of seroma, hematoma, wound dehiscence, local infection or sepsis and no need for wound dressing.	7, 14 days 8 weeks	Genta sponge	49	Perineal 1 (2)* Sacral 2 (4)	3 (6)	43 (88)	1 (2)	–
				Control	48	Perineal 5 (10)	2 (4)	36 (75)	0 (0)	–
Rosen et al. [27]	RCT	Primary wound healing = healing within 14 days without secretion or dehiscence/need for local treatment or dressing	14 days	Genta beads	22	Abscess 1 (5)	–	19 (86)^*^	1 (5)	20 ± 7^*,d^
				Control	22	Abscess 2 (9)	–	10 (45)	2 (9)	27 ± 12^d^
Moesgaard et al. [17]	RCT	Perineal wound infection (PWI) = presence of pus, either discharging spontaneously or requiring drainage Intra-abdominal abscess (IAA) = verified by surgical drainage or ultrasound-guided aspiration Septicemia (S) = positive blood culture	At discharge 1,3 months	Genta injection	41	PWI: 19 (46) IAA: 1 (2) S: 2 (5)	–	–	–	22 (10–100)^c^
				Control	38	PWI: 18 (48) IAA: 1 (2) S: 0 (0)	–	–	–	25 (10–72)^d^
Sachweh et al. [30]	CCS	–	–	Genta beads	80	10 (13)^*,a^	Persistent fistula 3 (4)^*^	70 (88)^*,b^	–	24^e^/16.6^*,f^
				Control	26	18 (69)	Persistent fistula 8 (31)	8 (31)	–	44
Mühleder et al. [19]	CCS	–	Daily during hospitalization	Genta beads	67	–	Persistent fistula 1 (1)	62 (93)^*^	–	–
				Control	42	–	–	23/39 (59)	–	–
		–	–							
Lütje et al. [15]	CS	Wound healing = no infection <14 days, no fever <3 days	–	Genta beads	25	–	–	23/24 (96)	–	20.4^g^

*RCT* randomized controlled trial, *CCS* comparative cohort study, *CS* cohort study, *PWI* perineal wound infection, *IAA* intra-abdominal abscess, *S* septicemia

^*^Statistically significant difference between the two study groups

^a^Continuous drainage 25 % versus intermittent drainage 2 %

^b^Continuous drainage 75 % versus intermittent drainage 97 %

^c^Median hospital stay (range)

^d^Mean hospital stay ± standard deviation

^e^Continuous drainage, distribution unknown

^f^Intermittent drainage, distribution unknown

^g^Distribution unknown

Perineal wound outcome was categorized as infectious complications, non-infectious complications, and primary wound healing for the purpose of this review (Table 3). Regarding infectious complications, three of six evaluable studies demonstrated a positive effect of local application of gentamicin. This significant difference was found in one out of four RCTs [22], and both comparative cohort studies [21, 25]. The impact on infectious complications was found in two out of three studies using gentamicin sponge [21, 22] and in one out of two studies using gentamicin beads [25].

Regarding non-infectious complications (i.e., seroma, hematoma, and dehiscence without infection), only two studies uniformly reported this outcome parameter. These two RCTs did not report a significant impact of gentamicin sponge on this perineal wound outcome parameter [20, 22]. One additional comparative cohort study reported a significant difference in the incidence of persistent perineal fistula in favor of gentamicin beads [25], although a fistula may be also considered as a long-term consequence of an initial infectious problem.

Primary perineal wound healing was the most consistently reported outcome parameter among seven included studies, including six comparative studies. However, the RCT of Collin et al. demonstrates that this outcome parameter is highly dependent on the time point of evaluation, ranging from 82 % after 7 days to 47 % after 1 month in the control group [20]. All three comparative cohort studies demonstrated a significantly higher percentage of primary perineal wound healing after local application of gentamicin [19, 21, 25], but only one out of three evaluable RCTs did show a positive effect of local gentamicin [23]. The positive effect of local gentamicin on primary wound healing was found in one out of three studies using sponges [21] and in all three comparative studies using beads [19, 23, 25]. Significant improvements in perineal wound outcome after local gentamicin were only observed in homogenous cancer populations, while the two RCTs containing subgroups of patients with IBD were both negative [20, 24].

### Reoperation and hospital stay

The reoperation rate for perineal wound infection was described in three studies and ranged between 2 and 5 % for the local application of gentamicin and ranged between 0 and 9 % when no topical antibiotics were applied [20, 22, 23]. Hospital stay was described in six studies (Table 3) [18, 20, 21, 23–25]. Out of five comparative studies, three studies reported a significant reduction in hospital stay (*p* < 0.05) [21, 23, 25]. The mean reduction in hospital stay was at least 7 days [21, 23, 25].

## Discussion

The present systematic review of the literature on perineal application of gentamicin after APR reveals that only a limited number of randomized and comparative cohort studies have been published, which are all of relatively low quality. Five of eight included studies were published more than 20 years ago. Most RCTs did not perform a power calculation and can be considered underpowered. Substantial heterogeneity was observed among the studies regarding underlying disease, surgical techniques, type of administration of gentamicin, antibiotic dosage, and peri-operative use of systemic antibiotics, definitions of outcome parameters, and timing of perineal wound evaluation. In addition, the non-randomized comparative studies have a substantial risk of bias. Taking all these methodological shortcomings in mind, we conclude that there is no convincing evidence for routine perineal application of gentamicin after APR. Statistically significant differences in favor of local gentamicin were mainly observed in comparative cohort studies, while the majority of RCTs were negative for both wound infection and primary wound healing.

In contrast to the present study, a recent systematic review showed that local application of gentamicin in all clean and clean contaminated wounds significantly reduces surgical site infections [26]. For patients undergoing colorectal surgery, several studies reported a positive effect of local gentamicin [10, 13]. However, gentamicin was mostly applied to the abdominal wound in these studies, without reporting on the use of topical antibiotics at the perineal wound with corresponding outcome. There are several potential reasons for a difference in wound healing between abdominal and perineal wounds, for example, with regard to the degree of bacterial contamination, tension on wound edges, and the received radioactivity dose in case of neo-adjuvant treatment [27, 28]. In addition, fluid may collect in the dead space above the closed perineum, and may become secondarily infected, with subsequent drainage through the perineum. The abdominal pressure on the perineal wound after (partially) resecting the pelvic floor may also negatively influence perineal wound healing. Because of these differences, our systematic review specifically focused on the perineal wound.

There are several patients and surgery-related factors influencing perineal wound healing after APR. Although the majority of patients were diagnosed with cancer, inflammatory bowel disease was also included in three studies. Inflammatory bowel disease patients significantly differ from cancer patients regarding perineal wound healing due to malnutrition, chronic pelvic inflammation, use of immunosuppressive medication, and pre-existing perineal fistulas and sinuses [29]. On the other hand, the use of pre-operative radiotherapy in rectal cancer patients may affect wound healing significantly [1]. Surgical techniques may also influence the occurrence of perineal wound infection and subsequent perineal wound healing. The omentum, for instance, is a major contributor to the local immune response and has the ability to promote angiogenesis [30, 31]. Therefore, placing an omental plasty in the pelvic cavity has been suggested to improve primary perineal wound healing [32]. Besides an omental plasty, several types of musculocutaneous or perforator flaps can be used for closure of the pelvic defect in selected patients undergoing extensive resection (i.e., exenterations) or salvage surgery for anal cancer [33–35]. None of the patients in the included studies underwent flap reconstruction, and the use of omental plasty was not specified. Therefore, the additive value of local gentamicin in combination with an autologous tissue flap or omental plasty is unknown. Furthermore, none of the included studies described laparoscopic surgery, the use of vessel sealing equipment, or patient positioning which have been shown to be related to blood loss, operative time, and risk of surgical site infection. These procedural characteristics may be of influence on perineal wound healing and may hamper extrapolation of results of the present systematic review to current clinical practice [24, 36].

Among the different application techniques, sponges, beads, and injection differ in local gentamicin concentration and release over time [4]. Besides differences in local antibiotic concentration, the collagen from which the gentamicin is delivered can have an influence on perineal wound healing itself [37]. This is most likely due to faster hemostasis and the protective effect on seroma and hematoma formation, which ultimately could affect primary perineal wound healing [37].

Gentamicin is most effective against gram-negative bacteria and some gram-positive bacteria, with the exception of anaerobe bacteria. Nelson et al. described in a systematic review of 182 RCTs that the addition of antibiotics against anaerobe bacteria in colorectal surgery reduces surgical site infections [38]. Based on these data, an antibiotic that is effective against anaerobic bacteria is currently most often being added as pre-operative prophylaxis [39]. However, antibiotics effective against anaerobe bacteria are most often not locally applied in a sustained matter and combined with gentamicin. Therefore, the local application of gentamicin in combination with an antibiotic targeting anaerobe bacterium might enhance the effectiveness against perineal wound problems.

In conclusion, a restricted number of low-quality randomized and non-randomized studies do not convincingly show a positive effect of local gentamicin on perineal wound infection or primary wound healing rate. APR for benign and malignant disease is essentially different, and treatment of distal rectal cancer has changed significantly since publication of most of the included studies. This underlines the need for new RCTs on locally applied gentamicin in more representative and homogeneous patient groups. At present, available literature does not support the routine use of local gentamicin application for perineal wound healing following APR.

## Appendix

See Table 4.

**Table 4:** Assessment risk of bias

Included Studies	Jadad et al. [14] (0–5)	Newcastle Ottawa quality assessment scale [38] cohort studies			
		Selection (0–4)	Comparability (0–2)	Outcome (0–3)	Total (0–9)
Collin et al. [7]	2	–	–	–	–
De Bruin et al. [8]	–	2	–	1	3
Gruessner et al. [11]	1	–	–	–	–
Rosen et al. [27]	1	–	–	–	–
Lütje et al. [15]	–	1	–	1	2
Sachweh et al. [30]	–	1	–	1	2
Mühleder et al. [19]	–	1	–	1	2
Moesgaard et al. [17]	2	–	–	–	–
